# Canine classical seminoma: a specific malignant type with human classifications is highly correlated with tumor angiogenesis

**DOI:** 10.1186/1471-2407-10-243

**Published:** 2010-05-28

**Authors:** Jong-Hyuk Kim, Chi-Ho Yu, Ji-Young Yhee, Keum-Soon Im, Na-Hyun Kim, Jung-Hyang Sur

**Affiliations:** 1 Department of Veterinary Pathology, Small Animal Tumor Diagnostic Center, College of Veterinary Medicine, Konkuk University, 1 Hwayang-dong, Kwangjin-gu, Seoul 143-701, Korea

## Abstract

**Background:**

Human seminoma is classified as classical seminoma (SE) and spermatocytic seminoma (SS). Human SE is known to be more malignant and metastasizing more frequently than SS. Tumor angiogenesis is highly related with tumor progression and metastasis, with microvessel density (MVD) being an important parameter of metastatic potential. Canine seminoma is not yet well-established as SE or SS type including correlation with angiogenesis. We classified canine SE and SS, and then compared them to tumor associated vessels.

**Methods:**

Twenty-three cases of canine seminomas (2 intratubular, 9 diffuse, and 12 intratubular/diffuse seminomas showing both intratubular and diffuse patterns) were classified as SE or SS by immunohistochemistry (IHC) using monoclonal antibody against PLAP and by PAS stain. The histopathological data were then compared to see if there was a correlation with SE or SS. Angiogenesis of seminomas were evaluated by immunohistochemical assay using polyclonal antibody against Von Willebrand factor (vWF) and by calculating the means of MVD, vessels area and perimeters using computerized image analysis. Statistical Package for Social Sciences (SPSS) program was used for various statistical analyses.

**Results:**

The numbers of PLAP+/PAS+ canine SEs were 8/23 (34.8%) and PLAP-/PAS- SSs were 15/23 (61.2%). All SE cases (8/8, 100%) were intratubular/diffuse types. SS types included 2 intratubular (2/15, 13.3%), 9 diffuse (9/15, 60%), and 4 intratubular/diffuse (4/15, 26.7%) types. MVD and vascular parameters in SEs were significantly higher than in SSs, showing the highest value in the intratubular/diffuse type. Seminomas observed with neoplastic cells invasion of vessels presented higher perimeter and area values than seminomas without conformed neoplastic cells invasion.

**Conclusion:**

In this study, we demonstrated a positive relationship between canine SE and tumor angiogenesis. Furthermore, we also showed that a tumor cells invasion of vessels were a correlated vascular parameter. Although metastasis of canine seminomas has rarely been reported, our results support that canine SE could have high metastatic potential similar to the human counterpart. Further studies are required to clarify the relationship between canine SE and clinical data with metastatic factors.

## Background

Testicular germ cell tumors are the most common tumor in male [1], and are classified as seminomas and nonseminomas [2]. Seminoma occurs frequently in the testicles of both human and canine [3-5]. Human seminomas are classified as classical (or typical) seminoma (SE) and spermatocytic seminoma (SS) according to the human World Health Organization (WHO) [6]. The two types are recognizable by placental alkaline phosphatase (PLAP) immunostaining and periodic acid-Schiff (PAS) staining [7]. Since SE originates from gonocytes [8], and gonocytes immunohistochemically express PLAP and carry PAS-positive intracytolplasmic granules [6], SE shows PLAP-positive immunostaining and PAS-positive staining [9,10]. Conversely, SS derived from mature spermatocytes [8], rarely expresses PLAP and is PAS-negative [9,10]

Human SE is usually found in young adults, but is rare in children and has high malignant and metastatic potential [4,11]. SS rarely metastasizes and occurs in old men [4,11]. Under microscopic examination, human SE consists of uniformly large cells and contain clear cytoplasm cells, and has abundant lymphocytes infiltration or granulomatous inflammation [12]. SS usually presents polymorphous neoplastic cells [13]. There are three cell types: large; intermediate; small (lymphocyte-like) cells. Each cell type has distinctive size and morphology [13,14]. SS shows more numerous mitotic figures than SE [15].

Canine seminoma is known to have low malignant behavior and rare metastases [16], although it displays malignant histological appearance, which is why canine seminoma has been regarded as human SS type [17]. However the classification of canine seminoma is not yet well-established, although some literatures have reported on canine SE and SS types [18,19].

Tumor angiogenesis is highly related to tumor progression, and a significant metastatic factor [20,21]. Angiogenesis promotes tumor growth by supplying essential oxygen and nutrition to neoplastic cells, and plays a role in the metastatic process, including invasion of tumor cells into microvessels [21-23]. Microvessel density (MVD; number of vessels per mm^2^) related tumor angiogenesis is regarded as a metastatic and prognostic indicator. Tumors which showed high malignancy and metastasis presented high MVD [24-26].

Some authors independently reported about canine SE and SS, and angiogenesis in seminomas [18,19,27], but no reports compared canine SE and SS to tumor angiogenesis. Therefore, the aims of this study were to examine the histopathological features and tumor angiogenesis of canine SE and SS, and to determine if there was a correlation with metastatic potentials.

## Methods

### Tissue specimens and clinical data

Twenty-three canine spontaneous seminoma tissues and their clinical data were collected from the Veterinary Medical Teaching Hospital of Konkuk University, Seoul, Korea or from private animal hospitals between 2003 and 2009. The animals at the time of surgery ranged in age from 3 to 14 years (mean 9.8 ± 3.19, two were unknown) and were of various pure or mixed breeds. All specimens were fixed in 10% neutral buffered formalin and subsequently embedded in paraffin wax. Sections of 4 μm thickness were cut from each tissue for staining with haematoxylin and eosin (HE) and immunohistochemistry (IHC). The samples were diagnosed in the Konkuk University Veterinary Medical Diagnostic Laboratory (Small Animal Tumor Diagnostic Center, Seoul, Korea).

### Histological examination

Serial 4 μm sections were acquired from each paraffin block and stained with HE and PAS. Histopathological diagnosis was based on light microscopy with HE staining. According to the WHO histological classification system for tumors of domestic animals [16], seminomas were classified as intratubular or diffuse. Intratubular/diffuse types were identified seminomas showing both intratubular and diffuse patterns [19].

### Immunohistochemistry

For immunohistochemical analysis monoclonal mouse anti-PLAP (DAKO, Glostrup, Denmark) and polyclonal rabbit anti-Von Willebrand factor (vWF; DAKO, Glostrup, Denmark) antibodies were used. Two-step Envision system-horse radish peroxidase (HRP; Dako REAL™ Envision kit; DAKO, Glostrup, Denmark) was applied for detection.

IHC was performed as described previously with a few modifications [28]. Briefly, slides were deparaffinized in xylene then hydrated in graded ethanol. Tissue sections were treated with 3% hydrogen peroxide (H_2_O_2_) solution for 20 minutes at room temperature, followed by washing phosphate-buffered saline (PBS, pH 7.4, 137 mM NaCl, 2.7 mM KCl, 10 mM Na_2_HPO_4_, 2 mM KH_2_PO_4_) three times. The antigens were retrieved by boiling the sections in Tris-EDTA buffer (pH 9.0) for 10 minutes in a microwave oven (650W). After cooling, the slides were washed three times in the PBS solution listed above. Subsequently, sections were incubated with anti-human PLAP antibody (1:50) in the refrigerator (4°C) overnight, and with polyclonal rabbit anti-human vWF antibody (1:600) for 1 hour and 30 minutes at room temperature. For secondary polymer, Envision system-HRP was used. The secondary polymer was applied to each slide for 40 minutes at room temperature, and the slides were washed with PBS 4 times. Next, the slides were incubated with substrates for Envision system-HRP until desired staining intensity developed. The color reaction was stopped by washing in distilled water twice, and counterstained with Harris hematoxylin. As positive control against anti-human PLAP was used a feline placenta section. As internal positive controls, smooth muscle cells of vascular walls and peritubular myoid cells were regarded. In negative control sections, we omitted primary antibodies, and replaced with PBS.

### Evaluation of MVD and vascular parameters

The method for measuring angiogenesis was used computerized image analysis by modifying previous studies [27,29,30]. MVD and vascular parameters, such as area and perimeter of microvessels were randomly assessed by choosing immunolabeled vessels with automated image analysis software (Image Pro Plus 5.1; Media cybernetics Inc., MD, USA). Digital images were acquired using a light microscope (Olympus; BX41, Dokyo, Japan) and digital image transfer software (Leica Application suite 2.7) at 400× magnification (40× objective and 10× ocular). Twenty fields of immunolabeled images were taken per tumor. Manual outlining of immunolabeled microvessels was performed, and then MVD and vascular parameters per square millimeter were calculated based on image analysis; vessels with a thick and muscular layer were not counted.

### Statistical analysis

The association between SE or SS types and categorical variables (histological type, tumor cells invasion of vessels, cryptorchid testis presence, lymphocytes infiltration, and presence of three cells type) were performed using the chi-square test respectively. The student's *t*-test and the analysis of variance (ANOVA) test were used for continuous variables. MVD and vascular parameters were correlated with SE or SS type and the presence of tumor cells invasion into vessels by the student's *t*-test and with histological types by ANOVA test. *p *< 0.05 was considered to be statically significant. Statistical analysis was performed using Statistical Package for Social Sciences (SPSS) v.11.0 program.

## Results

### Clinical and histopathological features

8 out 23 (34.8%) cases were identified in cryptorchid testes. The mean age of dogs with cryptorchid was 10.1 ± 3.4 years and without cryptorchid was 9.7 ± 3.2, respectively. There were not statistical significant between the presence of cryptorchidism and other histopathological features, such as histological type, lymphocytes infiltration, three cells type and tumor cells invasion of vessels. For histological classification, total 23 canine seminomas were classified as 2 intratubular (8.7%), 9 diffuse (39.1%), and 12 intratubular/diffuse seminomas (52.2%). Tumor cells invasions of lymphatic or blood vessels were observed in 12/23 cases (52.2%): intratubular 0/2 (0%), diffuse 3/9 (33.3%) and intatubular/diffuse 9/12 (75%) cases (*p *= 0.051). Lymphocytes were infiltrated in 13/23 (56.5%) cases. There was three cells type in 8/23 (34.8%) cases. Table 1 shows the characteristics of canine seminomas used in the present study.

**Table 1:** Characteristics of canine seminomas used in the present study.

Breed	Age (years)	Cryptorchid	Histological type	Tumor cells invasion	Lymphocytes infiltration	PLAP immunostaining	PAS staining
Cocker Spaniel	4	Absence	Intratubular	**×**	**×**	**-**	**-**

Yorkshire Terrier	13	Presence	Diffuse	**×**	**×**	**-**	**-**

Maltese	12.7	Absence	Diffuse	**×**	O	**-**	**-**

Yorkshire Terrier	13	Absence	Diffuse	**×**	**×**	**-**	**-**

Yorkshire Terrier	13	Presence	Diffuse	O	**×**	**-**	**-**

Maltese	9	Presence	Diffuse	O	O	**-**	**-**

Poodle	9	Absence	Intratubular/diffuse	O	**×**	**-**	**-**

Shih Tzu	unknown	Absence	Intratubular/diffuse	O	O	**+**	**+**

Maltese	10	Absence	Intratubular/diffuse	**×**	O	**+**	**+**

Yorkshire Terrier	6	Absence	Intratubular	**×**	**×**	**-**	**-**

Shih Tzu	6	Absence	Intratubular/diffuse	**×**	**×**	**+**	**+**

Maltese	12.5	Presence	Intratubular/diffuse	O	**×**	**+**	**+**

Maltese	12	Presence	Intratubular/diffuse	O	O	**+**	**+**

Akida	13	Absence	Diffuse	O	O	**-**	**-**

Maltese	9	Absence	Intratubular/diffuse	O	O	**-**	**-**

Maltese	7	Absence	Intratubular/diffuse	O	O	**+**	**+**

Yorkshire Terrier	12	Absence	Intratubular/diffuse	**×**	O	**-**	**-**

Maltese	10	Absence	Diffuse	**×**	O	**-**	**-**

Poodle	9	Presence	Intratubular/diffuse	O	O	**-**	**-**

Shih Tzu	9	Presence	Diffuse	**×**	O	**-**	**-**

Maltese	unknown	Absence	Intratubular/diffuse	O	**×**	**+**	**+**

Yorkshire Terrier	14	Absence	Diffuse	**×**	O	**-**	**-**

Shih Tzu	3	Presence	Intratubular/diffuse	O	**×**	**+**	**+**

Note: O, observed; **×**, not observed; +, positive; **-**, negative; PLAP, placental alkaline phosphatase; PAS, periodic acid-Schiff

### PLAP immunostain and PAS stain

**Figure 1:**
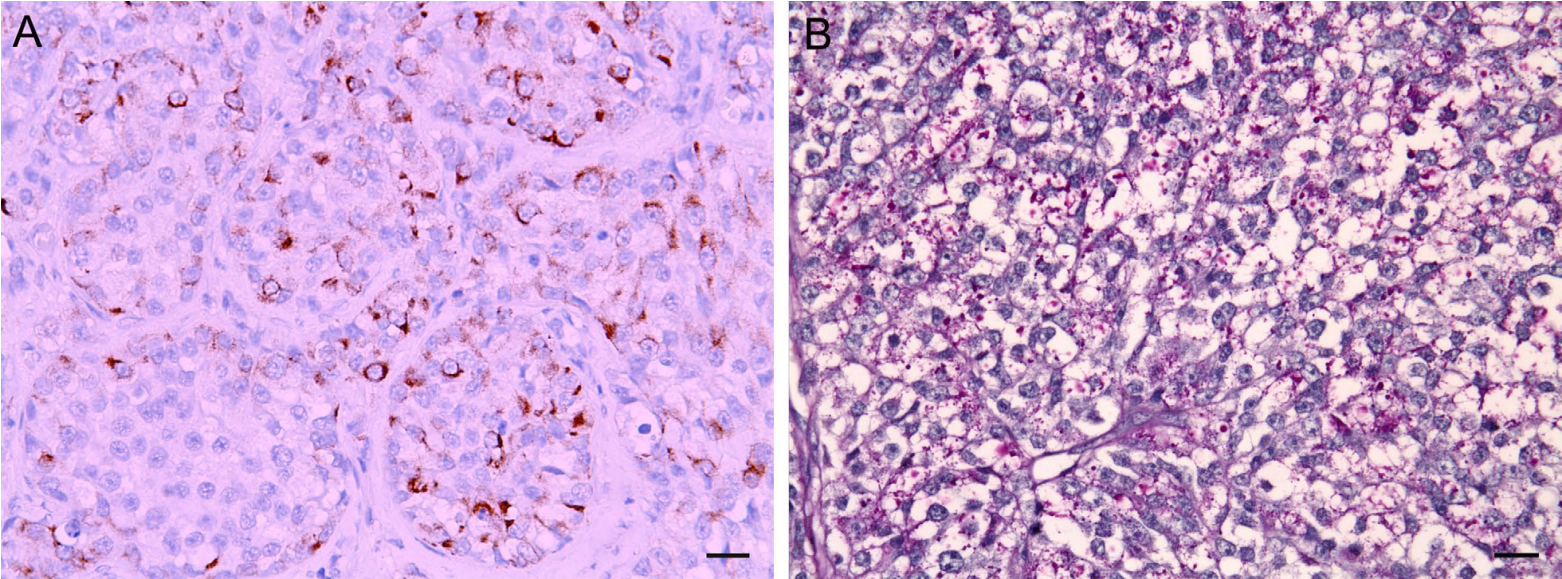
**Canine classical intratubular/diffuse seminoma**. Tumor cells in classical seminoma show PLAP-positive membranous and cytoplasmic immunostain and PAS-positive granule in cytoplasm. **A**. Immunoreaction of PLAP is brown with envision system-HRP. **B**. PAS-positive staining. Scale bar = 36 μm.

Canine seminomas analyzed by PLAP immunostaining and PAS staining of specimens resulted in 8 PLAP+/PAS+ seminomas (34.8%) and 15 PLAP-/PAS- seminomas (65.2%) cases (*p *= 0.004). PLAP+/PAS- or PLAP-/PLAP+ seminomas were not evident. Cytoplasmic staining of PLAP or PAS on tumor cells was considered to be positive (Figure 1A, B). On the bases of PLAP immunostaining and PAS staining pattern, 8 out 23 (34.8%) seminomas were classified as SE and 15 out 23 (61.2%) as SS. The mean age of dogs with SE was 8.4 years and with SS was 10.7 years. Canine SEs were all intratubular/diffuse types (8/8, 100%), while SSs were 2/15 (13.3%) intratubular, 9/15 (60%) diffuse and 4/15 (26.7%) intratubular/diffuse types (*p *= 0.004). Lymphocytes infiltration was observed in 4/8 (50%) of SEs and in 9/15 (60%) cases of SSs (*p *= 0.645). Tumor cells invasion of vessels was revealed in 6/8 (75%) SEs and 6/15 (40%) SSs (*p *= 0.110). All SEs presented a uniform cell pattern, and three cells type was displayed in 9/15 (60%) cases with SS (*p *= 0.011). Cryptorchid was occurred in 3/8 (37.5%) SEs and in 5/15 (33.3%) SSs (*p *= 0.842). Table 2 shows the relationship between tumor characteristics and SE/SS type analyzed by chi-square test.

**Table 2:** Correlation between tumor characteristics and canine classical or spermatocytic seminoma.

Variable		SE (*n *= 8)	SS (*n *= 15)	*P *value
**Histological type**	**Intratubular (*n *= 2)**	-	2	0.004
		
	**Diffuse (*n *= 9)**	-	9	
		
	**Intratubular/diffuse (*n *= 12)**	8	4	

**Lymphocytes infiltration (*n *= 13)**		4	9	0.645

**Cryptorchidism (*n *= 8)**		3	5	0.842

**Tumor cells invasion of vessels (*n *= 12)**		6	6	0.110

**Three cells type (*n *= 8)**		-	8	0.011

### MVD and vascular parameters

The values of MVD, perimeters, and areas were all calculated as means ± standard deviation (SD) per 1.6 mm^2^. MVD was 149.0 ± 64.0 in SE and 91.4 ± 16.9 in SS (*p *= 0.003). The perimeter was 11.5 ± 0.2 mm in SE and 7.2 ± 1.4 mm in SS (*p *= 0.009), while the area was 0.050 ± 0.013 mm^2 ^in SE and 0.028 ± 0.010 mm^2 ^in SS (*p *= 0.002).

**Figure 2:**
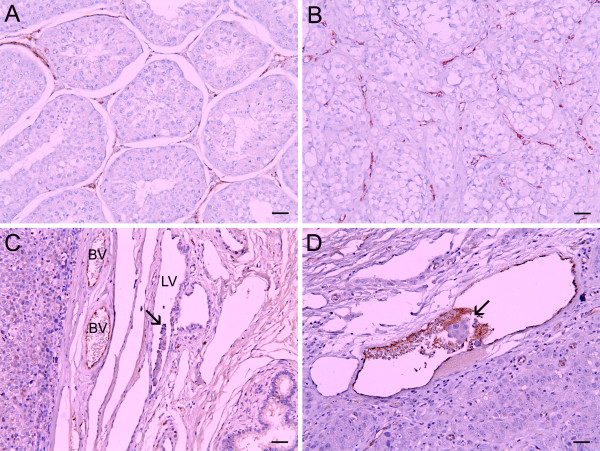
**Immunohistochemical staining with vWF to detect microvessels and tumor cells invasion into vessels in canine seminomas. A**. Microvessels in canine normal testis. **B**. Abundant microvessels were seen in intratubular/diffuse seminoma. **C**. Tumor cells invasion into lymphatic vessel is confirmed (arrow). **D**. Tumor emboli were seen in blood vessel (arrow). vWF is stained with envision system-HRP. Scale bar = 70 μm. BV = blood vessel; LV = lymphatic vessel.

According to the histological types, MVD was 90.0 ± 15.6 in intratubular, 87.9 ± 18.2 in diffuse, and 136.7 ± 57.7 in intratubular/diffuse types (*p *= 0.043). Perimeter was 5.6 ± 0.8 mm in intratubular, 7.1 ± 1.0 mm in diffuse, and 10.7 ± 3.3 mm in intratubular/diffuse types (*p *= 0.004). Area was 0.020 ± 0.006 mm^2 ^in intratubular, 0.030 ± 0.011 mm^2 ^in diffuse, 0.043 ± 0.016 mm^2 ^in intratubular/diffuse types (*p *= 0.030). Abundant microvessels were seen in intratubular/diffuse seminoma, while there were a few microvessels in normal testis (Figure 2A, B).

In addition, MVD and vascular parameters were analyzed according to the presence of tumor cells invasion into vessels. MVD was 126.1 ± 52.9 in seminomas with observed tumor cells invasion and 95.5 ± 37.0 in the cases with no tumor cell invasion into vessels (*p *= 0.122). The perimeter was 10.3 ± 3.2 mm and 6.9 ± 1.8 mm (*p *= 0.005), and area was 0.042 ± 0.016 mm^2 ^and 0.029 ± 0.012 mm^2 ^(*p *= 0.037) in tumor cell invasion and in no invasion cases, respectively. Immunohistochemical staining with vWF was performed to confirm tumor cells invasion into lymphatic or blood vessels in canine seminomas (Figure 2C, D). Table 3 presents the correlation of MVD and vascular parameters with histological type, PLAP/PAS stain and tumor cells invasion analyzed by the student's *t*-test or the ANOVA test.

**Table 3:** Correlation of microvessel density and vascular parameters with histological type, PLAP/PAS stain and microvessel invasion in canine seminoma.

Classification		MVD (number/1.6 mm^2^)	Perimeter (mm/1.6 mm^2^)	Area (mm^2^/1.6 mm^2^)
**Normal testis**		48	2.61	0.005

**Histological type**	**Intratubular Seminoma**	90.0 ± 15.6	5.6 ± 0.8	0.020 ± 0.006
	
	**Diffuse Seminoma**	87.9 ± 18.2	7.1 ± 1.0	0.030 ± 0.011
	
	**Intratubular/diffuse Seminoma**	136.7 ± 57.7	10.7 ± 3.3	0.043 ± 0.016

***P *value**		0.043	0.004	0.030

**PLAP/PAS stain**	**Classical Seminoma**	149.0 ± 64.0	11.5 ± 0.2	0.050 ± 0.013
	
	**Spermatocytic Seminoma**	91.4 ± 16.9	7.2 ± 1.4	0.028 ± 0.010

***P *value**		0.003	0.009	0.002

**Tumor cells invasion of vessels**	**Presence**	126.1 ± 52.9	10.3 ± 3.2	0.042 ± 0.016
	
	**Absence**	95.5 ± 37.0	6.9 ± 1.8	0.029 ± 0.012

***P *value**		0.122	0.005	0.037

Note: MVD = microvessel density

## Discussion

Human seminoma are classified as either SE or SS [6]. Most canine seminomas are similar to their human counterparts with fewer metastatic features [11,17,31]. However, results from the present study suggest the potential for highly metastatic tumor types among canine seminomas. Canine seminomas have been recently classified into SE or SS types [18,19]. Canine SE and SS were classified by PLAP immunostaining and PAS staining, and examined angiogenesis using computerized analysis in the present study. Finally, the correlation between SE/SS type and angiogenesis was evaluated. To the best of our knowledge, this study is the first report evaluating canine SE and SS types for angiogenesis. In the present study, the results suggested that canine SE demonstrate greater malignancy and metastatic potential than canine SS.

Canine SE and SS can both develop in older dogs. However, dogs with SS types were generally older, unlike human seminomas. These results are similar to those from a previous study reported by Grieco et al [19]. It is known that cryptorchidism develops more in human SE cases [4], although a similar association was not identified in the present study.

Under microscopic examination, canine SE/SS type was correlated with histological types (*p *= 0.004). SEs were all intratubular/diffuse types in this study. Purely intratubular and intratubular/diffuse types were observed more than the diffuse type in canine SE, whereas SSs were mostly diffuse types [19]. Intratubular/diffuse seminomas have previously demonstrated interstitial invasion by tumor cells. Other studies have reported that intratubular tumor types with signs of invasion were morphologically similar to human SEs, and that human SSs were related to intratubular and diffuse canine tumor types [18]. As our results support these, it may be correlated between canine SE/SS and histological type. However, further studies with the large number of canine seminoma cases are required to confirm the correlation. In addition, tumor cells invasion of lymphatic or blood vessels was mostly observed in intratubular/diffuse type, although this association was not significant.

Further, human SEs have demonstrated a relatively uniform cellularity, while human SSs show various sizes of tumor cells including large, intermediate, and small (lymphocyte-like) cells [6]. It has been observed canine SE and SS displayed these features consistent with human seminomas as well [18]. Maiolino et al reported that canine SS shows a proliferation of polymorphic neoplastic germ cells in their morphometric study [18]. Likewise, we presented that canine SS classified by PLAP immunostain and PAS stain showed various sizes of tumor cells (*p *= 0.011).

Moreover, a characteristic feature of human SE is the presence of a mature lymphocytes infiltrate, which is rarely observed in human SS [12]. Lymphocytes infiltration has been identified in some canine seminomas [32,33]. We also classified seminoma cases based on the presence of lymphocytes infiltrates. In our study, the presence of lymphocytes infiltrate was not different between canine SE and SS (*p *= 0.645), unlike human seminoma. There was no significant difference between the presence of lymphocytes infiltrates and histological types as well (*p *= 0.224). However, the relationship between lymphocytes infiltrate and the presence of three cells type were statistically significant; lymphocytes infiltration was frequently observed in seminoma cases with the presence of three cells type (*p *= 0.029).

The function of tumor-infiltrating lymphocytes (TILs) has not exactly been demonstrated in canine seminomas. However, Grieco et al has reported that TILs have a correlation with less malignant and metastasized characteristics in canine seminoma [32]. A direct correlation between TILs and canine SE or SS has not been established; however, the presence of three cells type was correlated with TILs in the present study. This suggests that TILs may be associated with canine SS play a role in canine SS because the three cells type is the feature of human SS. Further, our results suggested that canine SS might possess decreased malignancy and metastatic features than SE. Therefore, it was hypothesized that TILs play a role in less malignant canine seminoma as the report of Grieco et al stated [32]. Regarding this, further studies about the function of TILs in canine SE and SS are required.

Additionally, we investigated parameters related to tumor angiogenesis in canine SE and SS. Angiogenesis plays a critical role in the process of tumor growth and metastasis by supplying oxygen and nutrients to tumor cells [23,34]. Increased numbers of blood vessels is frequently associated with an increased risk of tumor metastasis [26]. In histological quantitative method, MVD is a robust prognostic and metastatic tool for tumor progression [25,35]. Restucci et al has previously examined MVD and vascular parameters in canine seminoma, but the study was not shown the differences between SE and SS [27]. Our results suggested that SEs were larger and harbored more vessels than SS; this suggested that canine SE may possess greater metastatic potential than canine SS. Furthermore, MVD, perimeters, and area values were highest in intratubular/diffuse type. This is concordant evidence, since we have shown that canine SE is correlated to the intratubular/diffuse type. In particular, the SDs of MVD in SE and in intratubular/diffuse seminoma revealed high values, 57.7 and 64.0 respectively (Table 3). These mean a significant variability of MVD in both SE and intratubular/diffuse type, and could suggest that there are areas alternating abundant microvessels with fewer vessels. This could mean a vigorous angiogenic reaction and be another parameter to present a high malignancy and metastatic potential.

In addition, tumor cells invasion into vessels could be an evidence of malignancy and a metastatic potential in canine seminoma [36]. The result of the present study demonstrated increased MVD and vascular parameters (areas and perimeter) in cases with tumor cells invasion into vessels. Both the mean and the SD of MVD in the cases with tumor cells invasion into vessels were higher than them in cases without that. Also, the means of vascular parameters, such as areas and perimeters were higher in the cases showing the presence of tumor cells invasion into vessels. These could indicate an active process for angiogenesis, and suggest that examination of tumor cell invasion into vasculature may serve as a valuable tool to evaluate malignancy and metastatic potential in canine seminomas and establish associations between angiogenesis, metastatic disease, and canine seminomas. Further studies are required to demonstrate associations between canine SE/SS type tumor angiogenesis and tumor cell invasion.

## Conclusion

Our results suggested that canine SE had greater malignant and metastatic potential than SS. The histological types were correlated with canine SE and SS types, with canine intratubular/diffuse types related to canine SE. This is the first report discussing angiogenesis in canine SE and SS. Increased MVD and vascular parameters in both canine SE and intratubular/diffuse type were examined, and the tumor cells invasion of vessels was correlated to increased vascular parameters. These results suggest that canine SE may have significant metastatic potential. Further studies are required to elucidate the relationship between canine SE and SS types and clinical data with metastatic factors.

## Abbreviations

ANOVA: analysis of variance; HE: hematoxylin and eosin; HRP: horse radish peroxidase; IHC: immunohistochemistry; MVD: microvessel density; PAS: periodic acid-Schiff; PBS: phosphate-buffered saline; PLAP: placental alkaline phosphatase; SD: standard deviation; SE: classical seminoma; SS; spermatocytic seminoma; TILs: tumor-infiltrating lymphocytes; WHO: World Health Organization.

## Competing interests

The authors declare that they have no competing interests.

## Authors' contributions

JH Kim participated in the design of this study, performed the statistical and image analysis, IHC experiment, and prepared the manuscript. CH contributed the statistical analysis, and JY supported image analysis. KS and NH supported the IHC experiment and the PAS staining, respectively. JH Sur designed this study, evaluated the IHC, and helped to draft the manuscript. All authors read and approved the final manuscript.

## Pre-publication history

The pre-publication history for this paper can be accessed here:

http://www.biomedcentral.com/1471-2407/10/243/prepub
